# 3D printing in neurosurgery education: a review

**DOI:** 10.1186/s41205-021-00099-4

**Published:** 2021-03-23

**Authors:** Grace M. Thiong’o, Mark Bernstein, James M. Drake

**Affiliations:** 1 Center for Image Guided Innovation and Therapeutic Intervention, Toronto, Canada; 2 Division of Neurosurgery, Hospital for Sick Children, University of Toronto, 555 University Avenue, Ontario M5G 1X8 Toronto, Canada; 3 Division of Neurosurgery, Toronto Western Hospital, University of Toronto, Ontario Toronto, Canada

**Keywords:** Additive Manufacturing, Neurosurgery Education, Rapid prototyping, 3D printing

## Abstract

**Objectives:**

The objectives of this manuscript were to review the literature concerning 3D printing of brain and cranial vault pathology and use these data to define the gaps in global utilization of 3D printing technology for neurosurgical education.

**Methods:**

Using specified criteria, literature searching was conducted to identify publications describing engineered neurosurgical simulators. Included in the study were manuscripts highlighting designs validated for neurosurgical skill transfer. Purely anatomical designs, lacking aspects of surgical simulation, were excluded. Eligible manuscripts were analyzed. Data on the types of simulators, representing the various modelled neurosurgical pathologies, were recorded. Authors’ countries of affiliation were also recorded.

**Results:**

A total of thirty-six articles, representing ten countries in five continents were identified. Geographically, Africa as a continent was not represented in any of the publications. The simulation-modelling encompassed a variety of neurosurgical subspecialties including: vascular, skull base, ventriculoscopy / ventriculostomy, craniosynostosis, skull lesions / skull defects, intrinsic brain tumor and other. Finally, the vascular and skull base categories together accounted for over half (52.8 %) of the 3D printed simulated neurosurgical pathology.

**Conclusions:**

Despite the growing body of literature supporting 3D printing in neurosurgical education, its full potential has not been maximized. Unexplored areas of 3D printing for neurosurgical simulation include models simulating the resection of intrinsic brain tumors or of epilepsy surgery lesions, as these require complex models to accurately simulate fine dissection techniques. 3D printed surgical phantoms offer an avenue for the advancement of global-surgery education initiatives.

## Introduction

Rapid prototyping has seen a rise in application since its infancy in the 1980’s [1]. It can be used to personalize health care through the process of fabricating patient-specific models [2]. This technology, also referred to as three-dimensional (3D) printing, when coupled with high-fidelity specifications or haptic feedback has led to the simulation of various intra-operative scenarios [3]. These simulations have in turn produced feedback data useful for face, construct and content validity of many models in different individual studies [4–9]. Additionally, validation studies have proven the resulting phantom applications in surgical training to be reproducible as well as to act as reasonable alternatives to cadaveric dissection [10–12].

The rapid adaptation of 3D printing technology in surgical training results in part from the conflict created by an increasing demand of surgical skill proficiency within a work-hour restricted environment [13]. The resulting simulators are a reasonable supplement for surgical apprenticeship. The intricacies of the surgical practice have seen an incorporation of phantoms in various surgical residency training programs, including neurosurgery [3, 14]. In the Low and Middle Income Countries (LMIC), there is a mismatch between the large neurosurgical volume and the much fewer number of neurosurgeons. Dewan et al. highlighted the gross disparity in the allocation of surgical workforce, that left large geographic treatment gaps, particularly in Africa [15]. The training of more surgeons has been proffered as a strategy to bridge this disparity [16]. Training of neurosurgeons through the traditional method of apprenticeship is not without its disadvantages, such as patient risk. 3D printed simulators overcome this shortcoming by presenting opportunities to equip trainees with common neurosurgical techniques as well as introducing complex skill sets to qualified neurosurgeons prior to carrying out live surgeries.

The core neurosurgical training sub-specialties are neuro-oncology, pediatric, functional, neurovascular, neuro-trauma, skull base and spine surgery. The shortcomings of technical skill transfer for these areas of specialization arising from cadaveric training, such as and more recently through on 3D printed surgical simulators.

The aims of this manuscript are to review the current global innovations of 3D printing in neurosurgical training, to identify both its global geographic distribution as well as the gaps in technical skill acquisition that 3D printing technology can potentially fill.

## Methods

A literature search was conducted on PubMed and Ovid for articles describing the design and application of 3D printing technology for neurosurgical training. The MeSH terms used were the following: “models / anatomic”, “neurosurgery education” and “printing, three dimensional”. The search concepts “models / anatomic”, “neurosurgery education” and “rapid prototyping” as well as “models / anatomic”, “neurosurgery education” and additive manufacturing” were also entered. PRISMA guidelines were applied for the identification, screening, eligibility checks and inclusion of relevant manuscripts [17]. Articles discussing phantoms of the brain, brainstem, intracranial vascular pathology and craniosynostosis were included. Those describing design and use of 3D printed surgical guides were likewise included, as were publications detailing approaches to the skull base. Manuscripts focusing purely on the neuroanatomy; those not tailored to a specific neurosurgical pathology, as well as those that lacked surgeon (or surgeon-in-training) task validation were excluded. Others discussing spine, and spinal cord phantoms, bioprinting, isolated virtual 3D, and non-human modelling were likewise excluded. Although spine surgery is an important part of neurosurgical training, its simulation focuses on skill sets that differ from those of brain and cranial vault neurosurgery.

Journals included in the final review were analyzed for demographic characteristics, trends and country affiliation of the authors. The texts were scrutinized to ensure that the countries where the phantoms were created and validated matched that of at least one of the authors. Once the final review process was completed, the extracted data were grouped using broad identifying terms: vascular, skull base, ventriculoscopy / ventriculostomy, craniosynostosis, skull lesions / skull defects, intrinsic brain tumor and other. Statistical analysis was performed using R (The R Foundation for Statistical Computing, Vienna, Austria), JMP Pro 14 (JMP Statistical Discovery™ from SAS, North Carolina, USA) and geolytics (Geolytics Pte Ltd, Singapore) software packages.

## Results

**Fig. 1:**
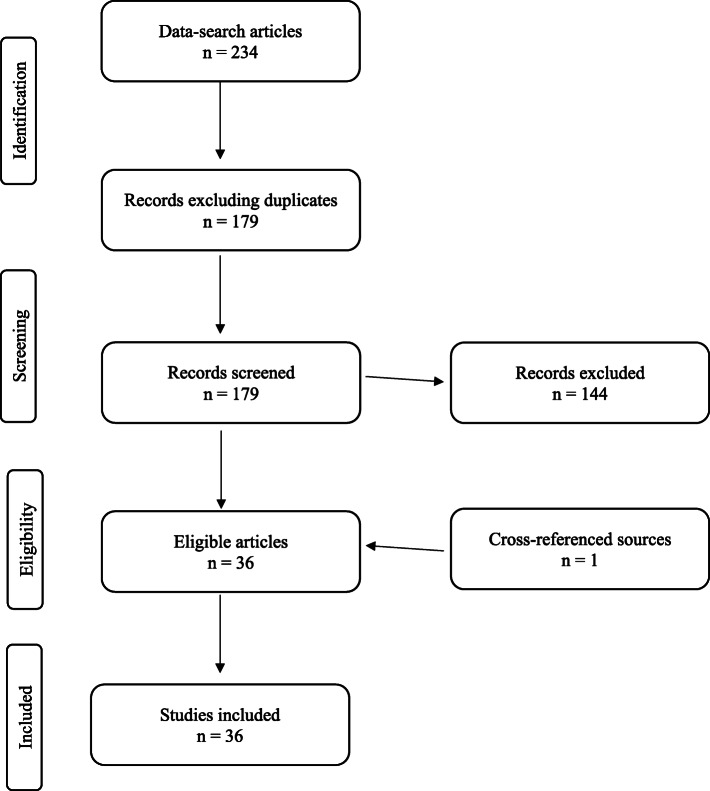
PRISMA search strategy summary

**Fig. 2:**
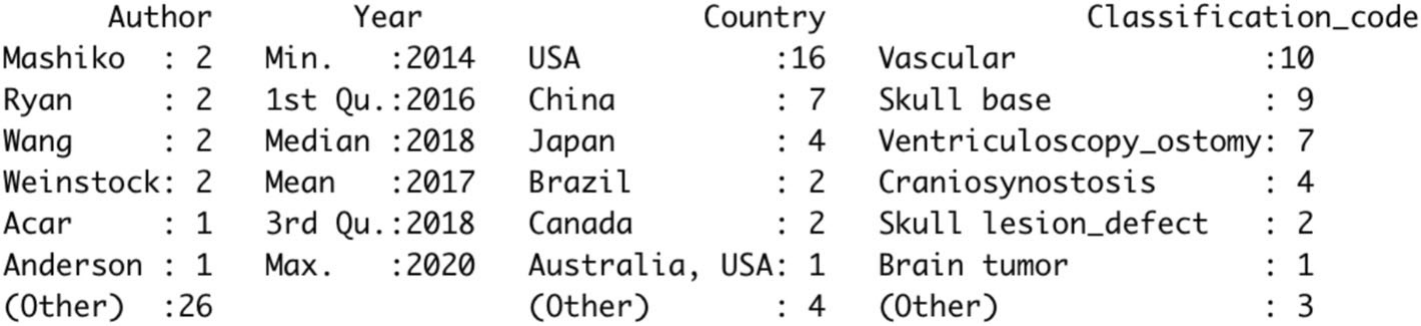
A tabulated summary of the distribution of the 3D printed phantoms identified in literature, corresponding to the author’s country affiliations

A summary of our search findings is illustrated in Fig. 1. A total of 36 articles representing ten countries were identified. Publications spanned the years between 2014 and 2020. In 2014, only one neurosurgery simulation manuscript was published, according to the outcome of the search criteria. In subsequent years, seven task-defining simulation phantoms were 3D printed in 2015, four in 2016, six in 2017, nine in 2018, seven in 2019 and two in the first 3.5 months of the year 2020. Figure 2 summarizes these findings in tabulated format.

Broad classifying terms grouped the inventions into seven domains: vascular, skull base, ventriculoscopy / ventriculostomy, craniosynostosis, skull lesions / skull defects, intrinsic brain tumor and other. Of the 36 simulators in our review, vascular models accounted for 10 and skull base models accounted for nine. The two categories, vascular and skull base together, account for over half (52.8 %) of the inventions. Ventriculoscopy / ventriculostomy was simulated in seven of the 36 publications (19.4 %), craniosynostosis in four of the 36 (11.1 %), skull lesions/defects in two of the 36 (5.5 %). Only one manuscript (2.8 %) simulated brain tumor surgery, which in this case was a patient-specific low-grade glioma resection. Single publications were also identified for epilepsy surgery (stereoelectroencephalography), congenital malformations (Sturge weber), and a final model that simulated brain retraction.

**Fig. 3:**
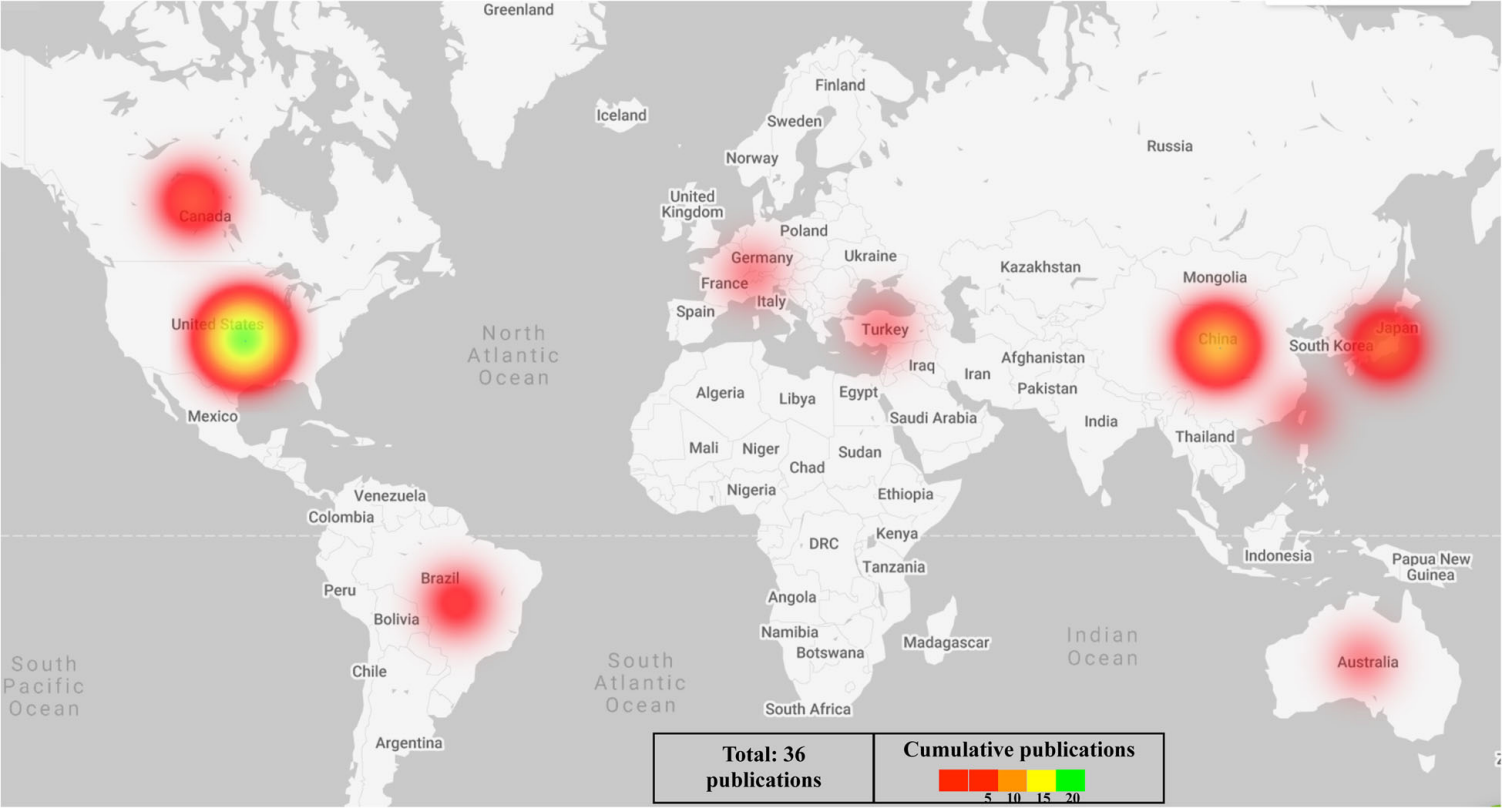
Geographic heat map illustrating the current distribution of 3D print technology’s use in neurosurgical education

**Fig. 4:**
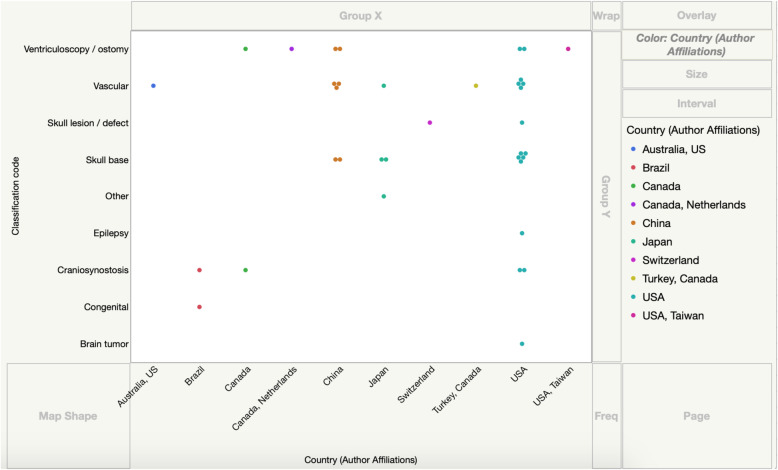
A graphical illustration of the disaggregated data by country of author’s affiliation

Of the 10 countries represented as having contributed to the data by virtue of author affiliation, publications from the USA alone accounted for 47.2 % of the total (17/36). China accounted for 19.4 % (7/36) of the simulated designs, Japan 11.1 % (4/36), Brazil and Canada each 5.6 % (2/36) and Switzerland and Netherlands each 2.8 % (1/36). Co-authored publications included Australia-USA, Turkey-Canada, Canada-Netherlands and USA-Taiwan; there was one article per collaborating author team. Finally, the resulting numeric data were used to generate a world geographic heat map (Fig. 3) as well as a graphical illustration of the categorical distribution of the simulated inventions by country/author affiliation (Fig. 4).

Disaggregation of the data yielded Fig. 4, a graph that illustrates the distribution of the various simulated design categories per author’s country of affiliation. Specifically, of the top three simulated categories vascular designs are clustered within China and the USA, skull base designs are clustered within China, Japan and the USA whereas ventriculoscopy designs have a fairly even distribution.

## Discussion

The key findings of this manuscript are the geographic depiction of 3D printing technology for neurosurgical education, the trends seen in the timeline between 2014 and 2019 and the simulated neurosurgical disease. By extension this highlights the areas that have as yet not been adequately simulated such as resective epilepsy surgery and brain tumor microdissection techniques.

The review found a general upward trend in the relevant publications, between the years 2014 to 2019, describing the design and neurosurgical application of skull and central nervous system (CNS) simulators. The projected inventions, therefore, for the year 2020 are expected to rise.

Phantoms simulating neurovascular and skull base surgical techniques are relatively common, according to literature. On the other hand, 3D printed models simulating resective epilepsy surgery and brain tumor microdissection techniques are not well described in the literature. [18,19]. This finding could indicate that 3D printing technology has not yet evolved to creating models that can simulate fine dissection detail. Currently, there exist a variety of soft materials for direct 3D printing such as TissueMatrix™ that can be used to fabricate quite complex designs using state of the art PolyJet technology, however, the high cost of manufacturing is a limitation. Additionally, despite the high physical fidelity of the resulting designs even this softest material lacks the appropriate tactile feel that would be ideal for simulating neurosurgical dissection. Perhaps not so surprising are the findings in the geographic heat map (Fig. 3), illustrating the paucity of 3D printing technology in the continent of Africa. The call for surgeons to engage in education on a global platform has been made [20, 21]. Our article introduces one way that the global-surgery education gap can be met through the versatile use of 3D printed surgical simulators. Introducing reusable simulators such as those validated for ventriculoscopy is a cost-effective strategy to train resident neurosurgeons both in the developing as well as the developed world. In higher income countries, where evolving surgical engineering research is feasible, developing new materials or re-inventing the use of existing materials which have higher neurosurgical functional fidelity could be explored.

In the developed world the clustering of inventions observed following disaggregation of our data (Fig. 4) is likely multifactorial. The divergence of biomedical engineering research to a surgical engineering focus would occur at different speeds within different institutions. In addition, it is possible that the observed clustering could be representative of the neuropathologic prevalence within each locality [22]. Consequently, the prevalence of disease would stimulate collaboration between surgeons and clinical engineers in the affected countries.

With regard to the representation of countries within North America all the Canadian publications meeting our inclusion criteria originated from the Center for Image Guided Innovation and Therapeutic Intervention lab (CIGITI) at The Hospital for Sick Children [5, 6, 23, 24].

## Conclusions

Overall, our review article argues that the already-proven benefit of 3D printing technology for surgical training could be the next frontier in global-surgery education.

## Data Availability

All data generated or analyzed during this study are included in this published article.

## Declarations

### Authors’ Information

All the authors are committed to finding innovative solutions to the global neurosurgery inequities.
